# Annual of the Universal Medical Sciences

**Published:** 1890-10

**Authors:** 


ANNUAL OF THE UNIVERSAL MEDICAL SCIENCES. A Yearly Report of the Progress of the General Sanitary Sciences Throughout the World. Edited by Charles E. Lyons, M. D., and seventy Associate Editors, assisted by over two hundred Corresponding Editors, Colaborators and Correspondents. Illustrated with Chromo-Lithographs, Engravings and Maps, in 5 Vols. 1890. F. A. Davis, Publisher. Philadelphia, New York, Chicago, Atlanta and London.

In the editor's preface it is stated that the improvements in this year's issue consist mainly in the creation of departments on subjects heretofore considered under general heads. Syphilis, for instance, under the editorship of Professor J. William White, of Philadelphia, appears as a special section, replete with information that could hardly otherwise be presented satisfactorily. Surgical Mycoses, edited by Prof. Grant Laplace, of Philadelphia, is another subject so treated, while that of Thoracic Surgery, by Prof. J. McFadden Gaston, of Atlanta, forms a special department, the value of which will become apparent.

Several sections will be found to have undergone modifications of value to the general practitioner. That of Oral Surgery, under Dr. R. Matos, of New Orleans, will be found to contain a review of the minor surgery of the teeth, limited in extent to the necessities of the physician. The department of Orthopædic Surgery, under Profs. Lewis A. and Reginald H. Sayre, of New York, has also been extended in scope, while that of Bacteriology, under Dr. Harold C. Ernst, of Boston, has received considerably more space. The section of Therapeutics, by Drs. J. P. Crozer Griffith and H. W. Cattell, also contains a resume of the therapeutical application of hypnotism.

All the improvements introduced into the 1889 issue have been continued. The general index has been made somewhat less bulky, the difficulty experienced in finding a desired head having rendered this advisable.

The subjects treated of in the five volumes embracing the medical and surgical advances, have been assigned to authors whose investigations into the literature of the several departments are presented concisely and yet clearly. The reader is rewarded by a full comprehension of the progress made in each branch, and the practitioner who desires a book of reference to refresh his mind upon any given subject, finds practical lessons of great utility under the different headings given in the general index. Few whose knowledge of progressive work is limited to the reading of text-books and medical journals, are prepared to understand what an immense field of inquiry is represented in these five volumes, containing, as set forth, a yearly report of the progress of the general sanitary sciences throughout the world. The references, appended in a condensed form, enable the searcher after truth to verify any statement in regard to which there may be doubt, and the prominence of a large proportion of the contributors affords a sufficient guarantee for the correctness of the data presented.

The excellence of the illustrations throughout the third and fourth volumes is in striking contrast to the paucity of embellishments in the other volumes, and though some of the matters treated of would have been elucidated by drawings, the first volume only contains two cuts, one delineating Dettwiler's spit-cups, and the other representing an unrecognized form of bacilli.

The typographical execution of the work reflects great credit upon the publisher, and the binding, in two styles, is tasteful, making quite an ornament to a medical library.

J. McF. G.

